# Successful Management of Atypical Hemolytic-Uremic Syndrome in Pregnancy Using Eculizumab: A Case Review

**DOI:** 10.7759/cureus.57973

**Published:** 2024-04-10

**Authors:** Faryal Altaf, Mikail Khanzada, Abeer Qasim, Anandu M Anto, Asim Haider, Misbahuddin Khaja

**Affiliations:** 1 Internal Medicine, BronxCare Health System, Bronx, USA; 2 Medicine, Lahore Medical and Dental College, Lahore, PAK; 3 Internal Medicine, St. Vincent Medical Center, Bridgeport, USA; 4 Internal Medicine/Pulmonary Critical Care, Icahn School of Medicine at Mount Sinai, BronxCare Health System, Bronx, USA

**Keywords:** atypical hemolytic-uremic syndrome (ahus), thrombocytopenia, microangiopathic hemolytic anemia (maha), thrombotic microangiopathy, plasmapheresis, pregnancy, monoclonal, eculizumab, hematology, hus

## Abstract

Hemolytic-uremic syndrome (HUS) is a rare thrombotic microangiopathy characterized by the triad of microangiopathic hemolytic anemia (MAHA), thrombocytopenia, and acute kidney injury. The disease is pathologically marked by fibrinoid necrosis within renal arterioles and glomerular capillaries. HUS can be categorized into typical variants, often linked to Shiga toxin-producing *Escherichia coli* (STEC) infection, and atypical variants that stem from dysregulation in the alternative complement pathway. Pregnancy is a recognized predisposing condition for HUS due to the potential reduction in complement regulatory proteins and the possibility of heightened maternal immune response. This report illustrates the case of a 36-year-old woman who, at 36 weeks of gestation, faced a breech presentation and was diagnosed with atypical HUS (aHUS) after placental abruption. Following a cesarean section, she developed complications, including a pelvic hematoma and bilateral hydronephrosis. Despite initial suboptimal response to plasmapheresis, the patient exhibited marked clinical improvement with eculizumab treatment, with no evidence of disease relapse.

## Introduction

Hemolytic-uremic syndrome (HUS) is a rare thrombotic microangiopathy characterized by microangiopathic hemolytic anemia (MAHA), thrombocytopenia, and acute kidney injury (AKI). Fibrinoid necrosis may also be seen in renal arterioles and glomerular capillaries [1]. HUS is classified into typical and atypical variants, commonly associated with Shiga toxin-producing *Escherichia coli* (STEC), and atypical variants due to alternate complement pathway dysregulation. Common triggers of atypical HUS (aHUS) include malignancy, autoimmune disease, infection, transplantation, and certain drugs [2]. Pregnancy is one such condition that may predispose an individual to HUS due to diminished complement regulator proteins being produced and the possibility of an exaggerated maternal response [3]. Most cases of pregnancy-induced HUS have occurred in individuals harboring mutations of membrane cofactor protein, factor H or factor I, all part of the alternate pathway.

While only affecting one in 25,000 pregnancies, HUS is mainly seen in the postpartum period and is associated with high rates of end-stage renal disease (ESRD), with up to 70% of ESRD patients requiring long-term dialysis [3,4]. Relapse of HUS has been seen in patients having undergone renal transplantation [4]. While plasma therapy has long been a mainstay of treatment, newer studies support the use of eculizumab, a monoclonal antibody that binds to and inhibits complement C5 [5]. Eculizumab has proven more effective in preventing long- and short-term renal outcomes and reducing relapse rates [6,7]. In this report, we detail the case of a 36-year-old woman who presented at 36 weeks of gestation with breech presentation and HUS triggered by placental abruption. The patient was admitted for a Caesarian section (C-section) with post-op complications of pelvic hematoma and bilateral hydronephrosis. The patient improved on treatment with eculizumab after poor response to plasmapheresis and showed no signs of relapse.

## Case presentation

We present the case of a 36-year-old female with a medical history of recurrent miscarriages, elective abortion, negative anti-phospholipid syndrome, chronic obstructive pulmonary disease (COPD), vitamin B12 deficiency, and a history of provoked pulmonary embolism (PE), who presented to the emergency department for a C-section following premature rupture of membranes (PROM) due to breech presentation. She had previously undergone bilateral tubal ligation three years prior. Other than her pregnancy, her physical examination was unremarkable. Post-C-section, she developed complications including pelvic hematoma and bilateral hydronephrosis. Severe anemia developed as a result of these complications, necessitating 15 blood transfusions, multiple platelet transfusions, fresh frozen plasma (FFP), and cryoprecipitate. To manage her condition, she underwent the placement of bilateral nephrostomy tubes and an inferior vena cava (IVC) filter. Due to the severity of her anemia and the need for transfusions, anticoagulation therapy was discontinued.

**Figure 1:**
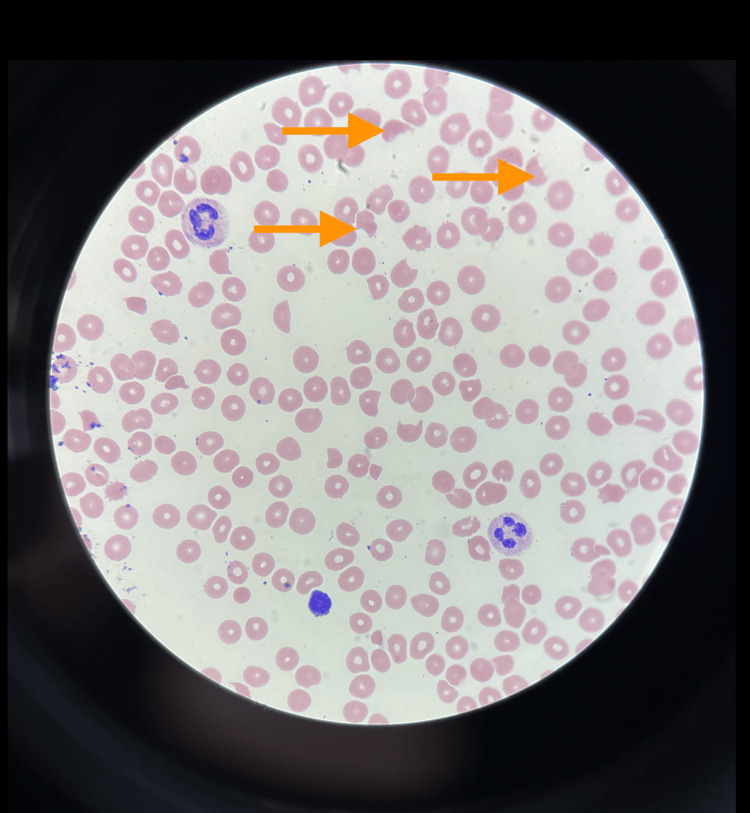
Peripheral smear showing schistocytes Arrow shows schistocytes

**Figure 2:**
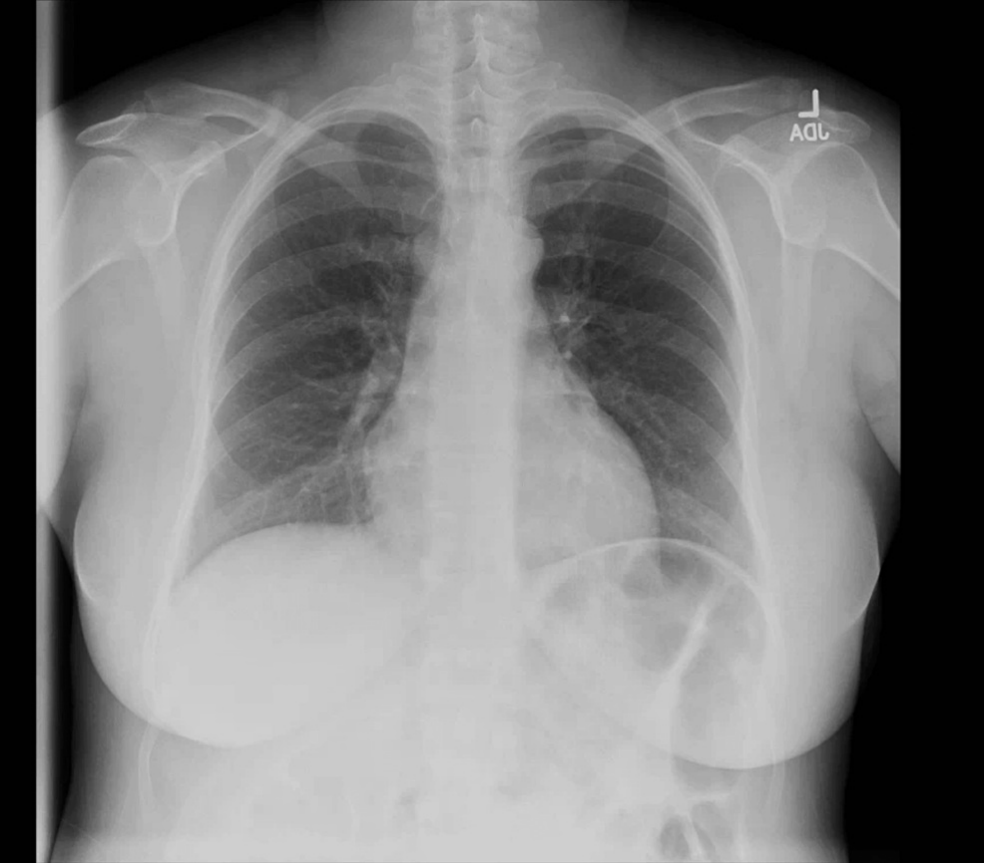
X-ray of the chest with no significant findings

The patient's laboratory tests indicated low haptoglobin levels. Tests for heparin-induced thrombocytopenia (HIT)/serotonin release assay (SRA) and direct and indirect Coombs were negative. Detailed results of the patient's laboratory tests can be found in Table 1. The patient's peripheral blood smear, presented in Figure 1, revealed the presence of schistocytes. In addition to these findings, the patient was experiencing hematuria, likely due to the nephrostomy tube, thrombocytopenia, and skin bruising. Additionally, a chest X-ray was performed, the results of which were normal, as can be seen in Figure 2. It is important to note that the patient has a history of a provoked PE from a year ago, which occurred during her last pregnancy following knee surgery and a long-distance flight. Following this event, a thorough thrombophilia workup was conducted to assess her risk of blood clots. This included tests for lupus anticoagulant, homocysteine, and anti-thrombin III which were all negative. 

**Table 1:** Initial laboratory values BUN: blood urea nitrogen; INR: international normalized ratio; APTT: activated partial thromboplastin clotting time; SRA: serotonin release assay; LDH: lactate dehydrogenase

Laboratory test		Reference values
Serum creatinine	6.1	0.50-1.10 mg/dl
BUN	78	8.0-26.0 mg/dl
Hemoglobin	7.1	12-16 g/dl
White blood cell count	8.9	4.8-10.8 k/ul
Platelet count	84	150-400 k/ul
Prothrombin time	14	9.9-13.3 seconds
INR	1.19	0.85-1.14
Total bilirubin	1.6	0.2-1.2 mg/dl
APTT	26	25.1-36.5 seconds
Coombs test	Negative	Negative
SRA	Negative	Negative
Direct bilirubin	0.5	0.0-0.3 mg/dl
LDH	2856	100-190 unit/l
Complement 3	147	90-150 mg/dl
Complement 4	30	16-47 mg/dl
G6PD	<10	30-200 mg/dl
ADAMTS13	81	68-163
D-dimer	10392	0-230 ng/ml
Fibrinogen	237	185-450 mg/dl
HIV ½ antibody	Negative	Negative

**Figure 3:**
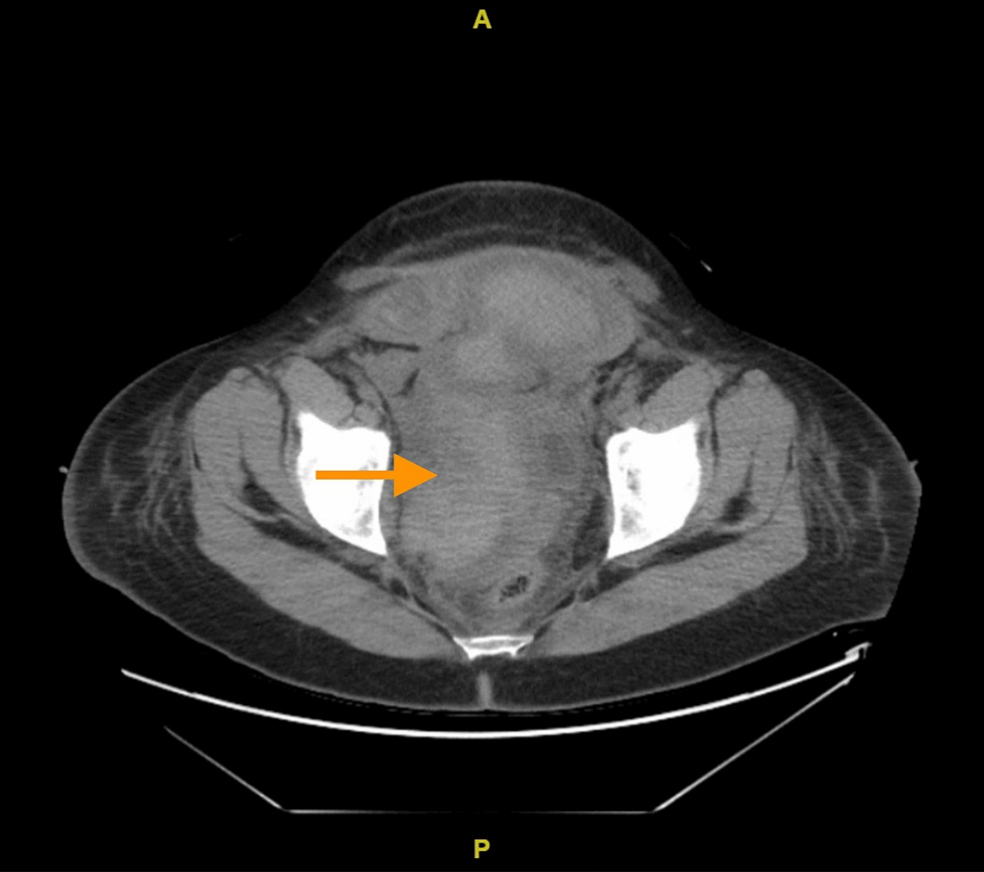
CT scan of the abdomen and pelvis showed bilateral nephrostomy tube placement with a resolution of hydronephrosis, renal cortical enhancement, and pelvic hematoma Arrow shows pelvic hematoma

**Figure 4:**
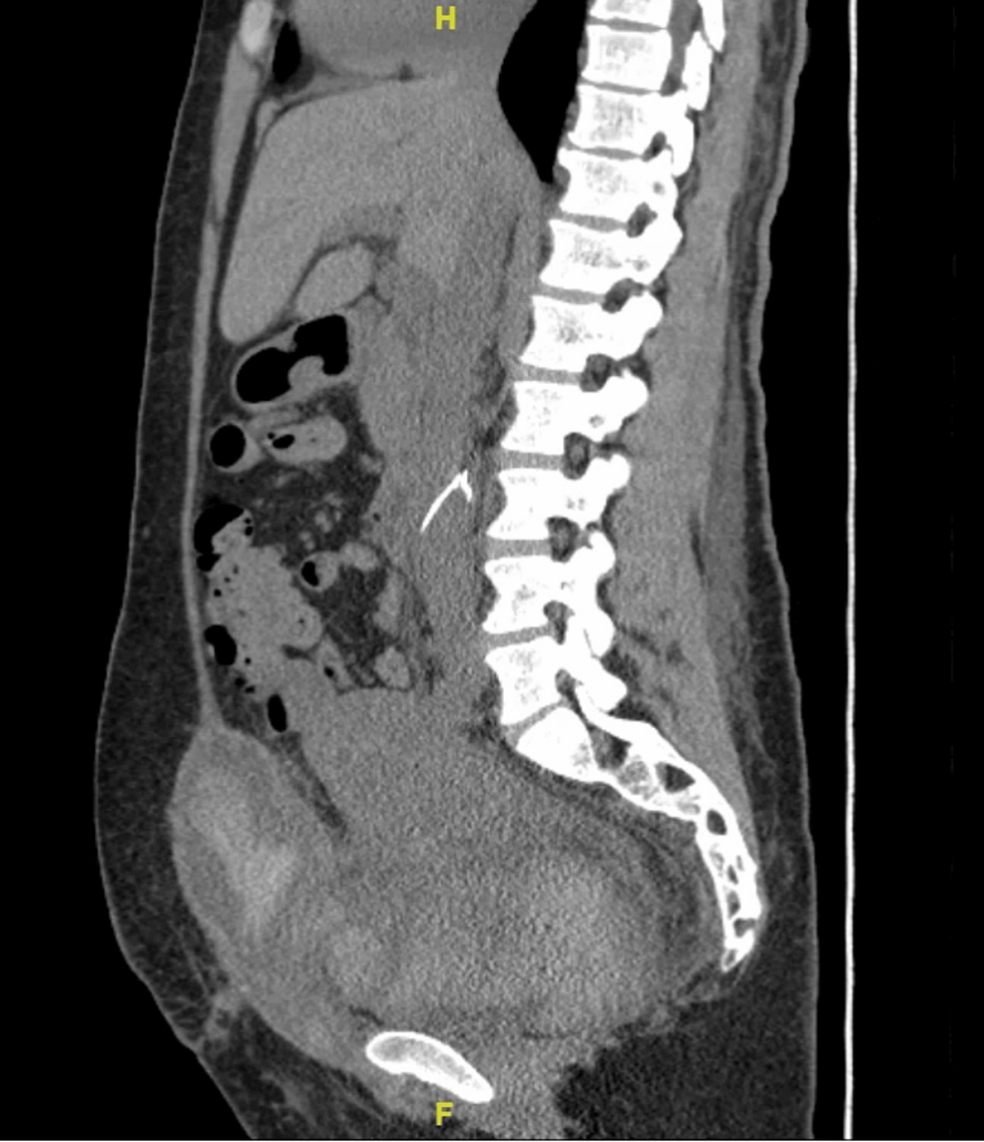
CT scan of the abdomen and pelvis showed bilateral nephrostomy tube placement with a resolution of hydronephrosis, renal cortical enhancement, and pelvic hematoma. Arrow shows pelvic hematoma

The patient underwent a CT scan of the abdomen and pelvis, which showed bilateral nephrostomy tube placement with a resolution of hydronephrosis, renal cortical enhancement likely acute tubular necrosis (ATN), given recent contrast, and pelvic hematoma as shown in Figure 3 and Figure 4. The patient had worsening renal failure, thrombocytopenia, and MAHA, leading to a significant concern regarding thrombotic microangiopathies. One week after the patient's presentation, a renal biopsy was done which showed acute diffuse thrombotic microangiopathy involving glomeruli and vessels. It also indicates acute tubular injury, interstitial fibrosis, and atrophy with moderate arteriolar thrombi and mucoid degeneration of arteries. The biopsy findings suggested eclampsia vs. aHUS.

The patient's differential diagnosis for MAHA includes thrombotic thrombocytopenic purpura (TTP), HUS, disseminated intravascular coagulation (DIC), as well as rheumatologic disease, hemolysis, elevated liver enzymes, and low platelets (HELLP), and drug-induced TMA. Normal levels of ADAMTS13 ruled out TTP. aHUS was on high differential due to renal failure, and renal biopsy supported this diagnosis. The patient was seen by hematology and diagnosed with pregnancy-induced aHUS. The patient's ADAMTS13 levels were within normal limits. Complements C3 and C4 and factor H were within normal limits. The patient had low vitamin B12 levels, for which intramuscular injections were started.

**Figure 5:**
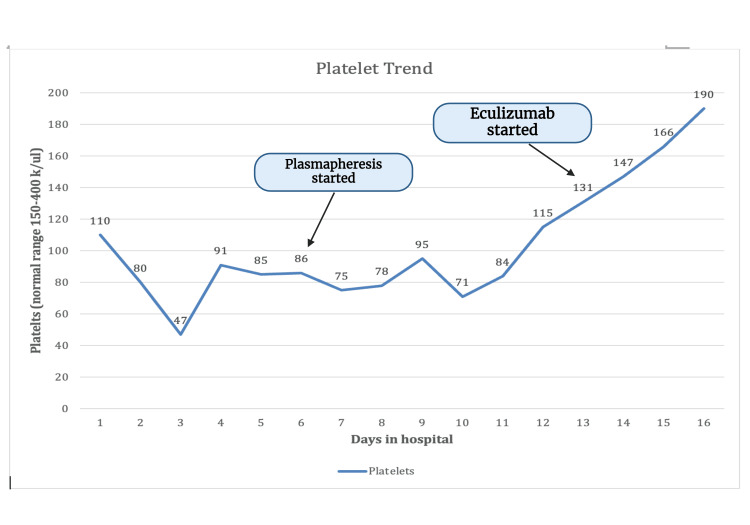
Trend of platelets during the hospital stay

**Figure 6:**
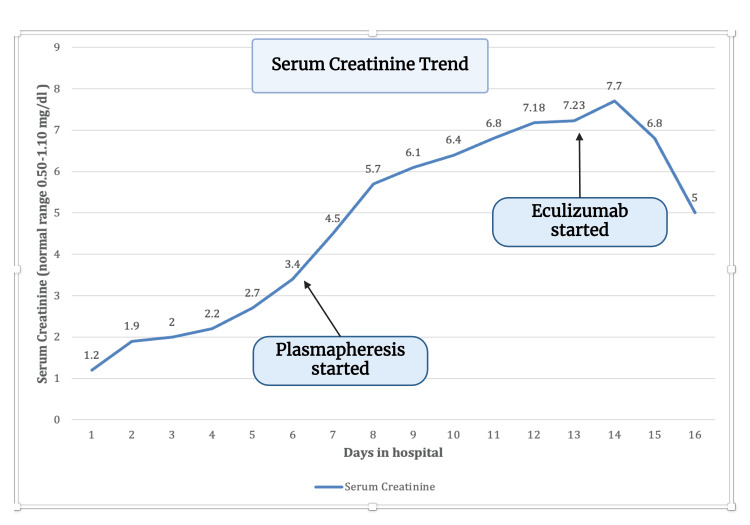
Trend of serum creatinine during the hospital stay

Without improvement, the patient was started on plasmapheresis on the fourth day of presentation for a total of five days. After plasmapheresis, the patient's platelets stabilized, but she continued to have schistocytes on the peripheral smear. The patient was transferred to a tertiary center for close monitoring. She was given the first dose of eculizumab two days after finishing plasmapheresis, which did not result in much improvement. The patient underwent a second dose of eculizumab, which resulted in massive improvement. The trend of platelets and creatinine is shown in Figure 5 and Figure 6. The patient's hemoglobin and platelet started to improve. Her creatinine, AKI, and potassium have begun to improve as well. Hydronephrosis improved on repeat imaging, and percutaneous nephrolithotomy (PCN) was removed after two weeks. The patient was discharged home with follow-up eculizumab doses to complete the course. The plan was to do eculizumab 900 mg weekly for four weeks and then 1200 mg on week 5, followed by 1200 mg every two weeks until seen by hematology and decided to stop. The patient was vaccinated against meningococcal with eculizumab due to the risk of meningitis.

## Discussion

We report a case of aHUS associated with pregnancy in the antepartum period in a 36-year-old woman who underwent C-section delivery due to breech presentation. In this instance, C-section delivery was further complicated by hydronephrosis and pelvic hematoma. While Fakhouri et al. found 80% of pregnancy-associated aHUS cases to occur in the postpartum period, our case is unique in that it occurred antepartum (36 weeks of gestation) [8]. Another study by Fakhouri et al. reported a mean time to diagnosis of 1.4 months, whereas our patient began treatment within six days [9].

Eculizumab has become more common in treatment protocols, primarily as a second- or third-line agent after intravenous (IV) corticosteroids, hemodialysis, and plasma exchange [10]. Plasma exchange remains the standard of care in cases where TTP, STEC HUS, or secondary microangiopathies haven't been ruled out [11]. It exerts a therapeutic effect by replacing deficient complement regulators and removing autoantibodies. Eculizumab has been found to lower rates of renal failure, dialysis, and death and to increase rates of disease remission [10]. While no protocols for eculizumab use in pregnant patients are available, the recommended dose for a 70 kg adult is 1200 mg twice weekly [12]. Adverse effects include an increased susceptibility to meningococcal infections, requiring patients to receive meningococcal vaccination two weeks before starting the drug; however, the benefits of treatment still outnumber the risks [13]. Treatment with longer-acting C5 inhibitors such as ravulizumab is recommended for six weeks with a lower treatment burden [14,15].

HUS is mainly a diagnosis of exclusion. In our patient, HUS was confirmed based on history and laboratory results. Our patient displayed all the hallmarks of HUS, including MAHA (4-7 schistocytes on peripheral smear and hemoglobin 7.1 g/dl), thrombocytopenia (platelets 84,000 per microliter), and signs of end-organ damage confirmed via a CT scan of the abdomen and pelvis.

Further confirmation of HUS came from the patient's normal ADAMTS13 score of <10. C3 complement levels are usually reduced; however, in this case, they were normal, making this an abnormal finding. A common finding in aHUS includes gene encoding factor H mutations. However, factor H levels in our patient were also normal [15,16]. Additional testing would be required to rule out genetic predisposition. While plasma exchange was started early, it failed to improve symptoms. The patient was then treated with eculizumab, which improved symptoms and remission [17,18].

It is unclear when to consider future pregnancies for our patient; measures such as a renal biopsy may be prudent to assess the degree of renal injury. Australian guidelines recommend a treatment duration of 24 months [16]. Considering complement regulation disorders are prevalent in 70% of HUS cases in pregnancy, genetic analysis such as exome sequencing should be considered to assess the risk of relapse and help plan future treatment strategies [15,19]. aHUS is a rare but life-threatening condition that should be considered in patients presenting with thrombocytopenia and acute renal injury. This report may help physicians consider the possibility of HUS in patients presenting with similar presentations in the antepartum period for timely diagnosis and treatment. 

## Conclusions

This case report highlights the critical role of timely recognition and intervention in the management of aHUS during pregnancy. The administration of eculizumab showed a remarkable improvement in the maternal condition without adverse fetal outcomes, underlining its potential as a lifesaving and effective treatment strategy. Clinicians must consider aHUS in the differential diagnosis of pregnant patients presenting with MAHA and renal impairment. Early consultation with a multidisciplinary team, including nephrology, hematology, and maternal-fetal medicine specialists, is vital to optimize outcomes. Moreover, this case adds to the growing body of evidence supporting the safety and efficacy of eculizumab in pregnancy, which may pave the way for more robust, future prospective studies. Ensuring the availability of eculizumab and developing protocols for its use in similar clinical scenarios could significantly improve maternal and fetal prognoses. We advocate for including eculizumab in treatment guidelines for aHUS in pregnancy, supported by further research to strengthen and validate our findings.
